# Body mass index and metabolic factors predict glomerular filtration rate and albuminuria over 20 years in a high-risk population

**DOI:** 10.1186/1471-2369-14-177

**Published:** 2013-08-20

**Authors:** Gabriele Nagel, Emanuel Zitt, Raphael Peter, Alfonso Pompella, Hans Concin, Karl Lhotta

**Affiliations:** 1 Agency for Preventive and Social Medicine, Rheinstraße 61, 6900 Bregenz, Austria; 2 Institute of Epidemiology and Medical Biometry, Ulm University, Ulm, Germany; 3 Department of Nephrology and Dialysis, Academic Teaching Hospital Feldkirch, Feldkirch, Austria; 4 Vorarlberg Institute for Vascular Investigation and Treatment, Academic Teaching Hospital Feldkirch, Feldkirch, Austria; 5 Department of Experimental Pathology, University of Pisa Medical School, Pisa, Italy

**Keywords:** Body mass index, Glomerular filtration rate, Albuminuria, Obesity, Gamma glutamyltransferase, Epidemiology

## Abstract

**Background:**

The number of individuals suffering from chronic kidney disease (CKD) is increasing. Therefore, early identification of modifiable predictors of CKD is highly desirable. Previous studies suggest an association between body mass index (BMI), metabolic factors and CKD.

**Methods:**

Data of 241 high risk patients with information on renal function and albuminuria from the Renal Disease in Vorarlberg (RENVOR) study (2010–2011) were linked with long-term measurements of metabolic factors in the same patients from the population-based Vorarlberg Health Monitoring & Prevention Program (VHM&PP) cohort study (1988–2005). Actual estimated glomerular filtration rate (eGFR) and urinary albumin creatinine ratio (ACR) were determined. BMI, blood pressure, fasting glucose, total cholesterol, triglycerides and Gamma-glutamyltransferase (GGT) were available from previous health examinations performed up to 25 years ago. Linear regression models were applied to identify predictors of current renal function.

**Results:**

At all-time points BMI was significantly inversely associated with actual eGFR and positively with actual albuminuria in men, but not in women. Serum GGT and triglycerides were significantly positively associated with albuminuria in men at all-time points. Fasting glucose levels more than 20 years earlier were associated with increased albuminuria in women and reduced eGFR in men, whereas at later time points it was associated with albuminuria in men.

**Conclusions:**

BMI, serum GGT, and triglycerides are long-term predictors of renal function in men. In women however, anthropometric and metabolic parameters seem to be less predictive of eGFR and albuminuria.

## Background

According to the World Health Organisation (WHO) obesity is one of the greatest public health challenges in the 21st century. Obesity causes 2-8% of health costs and 10-13% of deaths in Europe [1]. Not only is obesity a well-known risk factor for diabetes, cardiovascular disease and cancer, but it is also increasingly being recognised as contributing to the development of chronic kidney disease (CKD). According to a Swedish study, 16% of chronic renal failure cases in men and 11% in women can be attributed to obesity [2]. A recent study of over a million individuals showed that overweight and obesity in adolescence increased the risk for end-stage renal failure three- and sevenfold 25 years later [3].

The metabolic syndrome (MetS) is a cluster of different metabolic risk factors, such as obesity, hypertension, insulin resistance/hyperglycaemia and dyslipidaemia [4]. Obesity is linked to the metabolic syndrome, which is clearly associated with CKD [5]. Beyond the metabolic factors (elevated fasting glucose, hypertriglyceridemia and low high-density lipoprotein (HDL) cholesterol) and hypertension included in the definition of the metabolic syndrome, serum levels of the enzyme gamma glutamyltransferase (GGT) have been described as a risk factor for CKD [6]. There is recent evidence that non-alcoholic fatty liver disease is associated with increased risk of CKD [7]. However, very little is known about the patterns of metabolic factors associated with glomerular filtration rate (GFR) and albuminuria over time.

The aim of the present retrospective study was to assess the long-term associations between body mass index (BMI), blood pressure, blood glucose, total cholesterol and GGT levels, on the one hand, and estimated glomerular filtration rate (eGFR) and albuminuria in high-risk patients, on the other hand. To do this, we linked results from a recent cross-sectional study in patients at risk for kidney disease with data that had been collected at general health examinations of the same patients during the Vorarlberg Health Monitoring and Prevention Programme up to 25 years ago.

## Methods

### Cross-sectional study

Between June 2010 and August 2011 patients at high risk for kidney disease aged between 40 and 70 years were offered kidney disease screening at a general practitioner’s office in Vorarlberg by the Agency for Preventive and Social Medicine as part of the RENal diseases in VORarlberg (RENVOR) study. High risk was defined as the presence of one or more of the following disease conditions: existing diagnosis of diabetes mellitus type 2 according to the WHO definition, blood pressure above 140/90 mm Hg or being on antihypertensive medication, or established diagnosis of atherosclerotic cardiovascular disease (CVD). Data on current smoking status, onset of diabetes mellitus, hypertension or cardio-vascular disease, comorbidity and medication were collected by questionnaires, which had been filled in by the treating physicians. In addition, height, weight, waist and hip circumference were measured, and blood as well as urine samples were collected. Fasting plasma glucose, total cholesterol, triglycerides, LDL cholesterol, HDL cholesterol, GGT and C-reactive protein (CRP) were measured with standard methods. Creatinine was determined by the Jaffe reaction calibrated to IDMS standard. For calculation of the eGFR the CKD-EPI formula was applied. The urinary ACR was determined in a spontaneous morning urine sample.

The study was performed in accordance with the Declaration of Helsinki. All participants gave full informed consent.

### Longitudinal study

The RENVOR dataset was merged with data from previous health examinations held by the Vorarlberg Health Monitoring and Prevention Programme (VHM&PP). VHM&PP has been described in detail previously [8]. In brief, the VHM&PP was set up in 1970 as a population-based surveillance/screening programme for the prevention of chronic diseases such as cardiovascular disease and cancer. The program included a thorough clinical examination of the participants. Furthermore, height and weight were measured with the participants wearing light indoor clothes and no shoes. Systolic and diastolic blood pressure was measured in a seated position. Information on smoking status was recorded, categorizing the participants in “ever smokers” or “never smokers”. A blood sample for determination of fasting glucose, total cholesterol, triglycerides and GGT levels was taken. At each examination informed consent to store and process the data was obtained from all participants. Until now the program has collected information on cardiovascular risk factors in more than two-thirds of the population of Vorarlberg, the western-most state of Austria with approximately 370.000 inhabitants. This study was approved by the Ethics Committee of the State of Vorarlberg.

**Figure 1:**
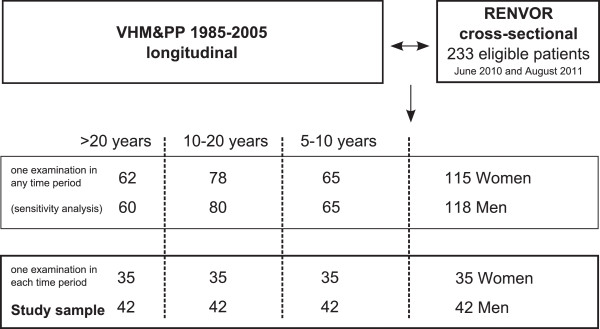
Selection of study population.

For a subset of the RENVOR patients data from regular previous health examinations (VHM&PP) were available. Only participants, who had attended at least one health examination in each time period (5–10 years, 10–20 years, >20 years) prior to the cross-sectional RENVOR study, were selected for further investigation. If more than one examination per time period was available, the examination closest to the time point 7.5 years, 15 years and 25 years was chosen. This procedure revealed 77 individuals with four measurements each (231 previous, 77 current) over a period of 25 years (Figure 1).

### Statistical analysis

Estimated GFR and the urinary ACR served as outcome parameters for linear regression models. We transformed ACR using the natural logarithm as ACR was severely skewed. Models with both dependent and independent variables log transformed are interpreted as percent change in ACR, while the independent variable (e.g. log GGT) changes by one percent. Models were stratified by sex and adjusted for age. Models were additionally adjusted for smoking status (data not shown). We repeated the analyses including also individuals who had measurements in any but not in each of the time intervals.

We calculated a sum score of pathologically elevated risk factors from longitudinal VHM&PP health examination data containing BMI, systolic blood pressure, plasma glucose, total cholesterol, triglycerides and GGT using the following cut-off points: >30 kg/m^2^, >140 mmHg, >5.6 mmol/l, >5.2 mmol/l, >2.0 mmol/l, and >18 U/l in women, >28 U/l in men for GGT, respectively. The sum score was calculated for each time period prior to commencement of the cross-sectional RENVOR study. An overall score was calculated as the mean of the sum scores for each time period.

Associations between the sum score and ACR, eGFR were modelled as in preceding analyses using linear regression adjusted for age and stratified by sex. Sex differences were investigated via inclusion of an interaction term of sex and the sum score.

All calculations were performed with the statistical software package SAS, release 9.2 (SAS Institute, Cary, NC; USA).

## Results

### Characteristics of the study population

A total of 241 patients with an established diagnosis of diabetes mellitus, hypertension or cardiovascular disease (CVD) were recruited by physicians in the RENal diseases in VORarlberg (RENVOR) study. Due to missing questionnaire data eight patients were excluded, leaving 233 (115 women and 118 men) for the present analysis. The baseline characteristics of all RENVOR participants are shown in Table 1. Mean age at recruitment was 60.4 years. Of the participants 80.2% suffered from hypertension, 36.5% from diabetes and 16.7% from CVD. The prevalence of an eGFR < 90 ml/min/1.73 m^2^ was 33% and of an eGFR < 60 ml/min/1.73 m^2^ 5.6%. An ACR >30 mg/g was detected in 17% of the patients and an ACR >300 mg/g was observed in eight (3.4%) of the patients. In the RENVOR study only 3 persons met the criteria (eGFR < 60 ml/min/1.73 m^2^ and ACR > 30) for CKD.

**Table 1:** Characteristics of RENVOR study population

		**RENVOR study population (n = 233) **^**1**^		**Subset with regular previous health examinations (n = 77)**	
		**Women**	**Men**	**Women**	**Men**
N (%)		115 (49.4%)	118 (50.6%)	35 (45.5%)	42 (54.5%)
Age (years), median (Q1, Q2)		60.6 (54.5, 66.9)	60.3 (52.2, 64.9)	60.7 (51.5, 67.0)	62.8 (57.9, 66.4)
Smoking status	Ever smoker	38 (33.0%)	68 (57.6%)	13 (37.1%)	27 (64.3%)
Disease status	Diabetes Mellitus	38 (33.0%)	47 (39.8%)	7 (20.0%)	13 (31.0%)
	Hypertension	96 (83.5%)	91 (77.1%)	27 (77.1%)	29 (69.1%)
	Cardiovascular disease	12 (10.4%)	27 (22.9%)	1 (2.9%)	11 (26.2%)
Time since first diagnosis of underlying disease^2^ (years), (N = 190), median (Q1, Q3)		8.0 (2.0, 14.0)	6.0 (2.0, 12.0)	7.5 (2.5, 15.0)	6.0 (3.0, 13.0)
**Laboratory parameters [median (Q1, Q3)]**					
WHR		0.87 (0.84, 0.92)	0.97 (0.92, 1.02)	0.86 (0.83, 0.92)	0.96 (0.90, 1.02)
BMI (kg/m^2^)		27.5 (24.5, 31.6)	29.4 (26.1, 32.9)	25.5 (23.5, 28.6)	27.0 (25.4, 31.8)
Total cholesterol (mmol/l)		5.24 (4.73, 6.09)	5.00 (4.19, 5.75)	5.32 (4.73, 6.36)	5.02 (4.22, 5.74)
LDL cholesterol (mmol/l)		2.46 (1.97, 2.97)	2.30 (1.78, 2.90)	2.35 (1.91, 3.00)	2.34 (1.86, 2.87)
HDL cholesterol (mmol/l)		1.32 (1.16, 1.58)	1.16 (0.96, 1.37)	1.45 (1.19, 1.63)	1.20 (0.98, 1.42)
Triglycerides (mmol/l)		1.48 (1.00, 1.90)	1.55 (1.12, 2.30)	1.42 (0.95, 2.04)	1.54 (1.10, 2.10)
Gamma-GT (units/l)		19.0 (13.0, 32.5)	36.0 (24.0, 57.0)	19.0 (13.0, 63.0)	32.0 (24.0, 62.0)
CRP (mg/dl)		0.3 (0.1, 0.6)	0.2 (0.1, 0.3)	0.2 (0.1, 0.6)	0.1 (0.1, 0.3)
eGFR (ml/min/1.73 m^2^)		95 (85, 102)	95 (87, 102)	95 (85, 100)	94 (88, 101)
<90 ml/min/1.73 m^2^	N (%)	42 (36.5%)	35 (29.7%)	12 (34.3%)	11 (26.2%)
<60 ml/min/1.73 m^2^	N (%)	9 (7.8%)	3 (2.5%)	2 (5.7%)	1 (2.4%)
Albumin/creatinine ratio (mg/g)		7.91 (4.64, 16.0)	6.29 (3.53, 19.2)	6.92 (4.64, 13.3)	7.10 (3.52, 16.0)
>30 mg/g	N (%)	17 (14.9%)	23 (19.5%)	2 (5.9%)	6 (14.3%)
GFR < 60 ml/min/1.73 m^2^ or ACR > 30 mg/g	N (%)	24 (21.1%)	24 (20.3%)	3 (8.8%)	7 (16.7%)

^1^ Questionnaire missing for 8 participants.

^2^ onset of diabetes mellitus, hypertension or cardio-vascular disease.

Abbreviations: *BMI* body mass index, *WHR* waist -to-hip ratio, *CRP* C-reactive protein, *eGFR* estimated glomerular filtration rate.

For 77 participants (35 women with mean age 60.0 (SD 7.8) years and 42 men with mean age 60.9 (SD 7.0) years) in the RENVOR study a complete dataset of previous VHM&PP examinations in the pre-specified time periods (233 investigations in total) was available. These 77 patients comprised the study population for the 25-year retrospective observational study.

### Cross-sectional study

In general, anthropometric and metabolic parameters were seen to have only weak associations with eGFR and ACR in the cross-sectional setting. The results of linear regression analyses are presented in detail in Table 2. In women, a significantly inverse association between waist-to-hip ratio (WHR) and eGFR was observed, while in men BMI and CRP levels were seen to be inversely related to eGFR.

**Table 2:** **Cross-sectional associations between anthropometric/ blood parameters and eGFR and ACR**^**1 **^**by sex**

	**eGFR**	**ACR**
	**Beta (95% CI)**	**Beta (95% CI)**^**1**^
	**Women**	
Total N	115	114
WHR*10	* -3.89 (−7.37, −0.41)	−0.01 (−0.31, 0.29)
BMI [kg/m^2^]	0.19 (−0.22, 0.60)	−0.00 (−0.04, 0.03)
Total cholesterol [mmol/l]	−1.46 (−3.87, 0.95)	* -0.23 (−0.43, −0.03)
LDL cholesterol [mmol/l]	−0.70 (−4.09, 2.68)	* -0.31 (−0.59, −0.03)
HDL cholesterol [mmol/l]	−5.17 (−12.45, 2.11)	−0.51 (−1.13, 0.10)
Log(Triglycerides) [mmol/l]	2.92 (−2.95, 8.80)	* 0.60 (0.12, 1.08)
Log(Gamma-GT) [units/l]	−2.14 (−5.24, 0.96)	0.13 (−0.14, 0.41)
Log(CRP) [mg/dl]	−0.70 (−3.51, 2.10)	−0.15 (−0.39, 0.08)
	**Men**	
Total N	118	118
WHR*10	−0.56 (−3.93, 2.81)	0.27 (−0.14, 0.68)
BMI [kg/m^2^]	* -0.69 (−1.30, −0.08)	* 0.06 (0.00, 0.13)
Total cholesterol [mmol/l]	0.85 (−1.35, 3.04)	−0.11 (−0.38, 0.16)
LDL cholesterol [mmol/l]	1.10 (−2.02, 4.21)	−0.29 (−0.67, 0.09)
HDL cholesterol [mmol/l]	5.42 (−2.57, 13.42)	−0.31 (−1.31, 0.69)
Log(Triglycerides) [mmol/l]	−2.92 (−7.31, 1.47)	0.23 (−0.32, 0.77)
Log(Gamma-GT) [units/l]	−0.96 (−4.21, 2.28)	0.07 (−0.34, 0.47)
Log(CRP) [mg/dl]	* -2.69 (−5.31, -0.07)	0.20 (−0.12, 0.53)

Models are adjusted for age.

* Significant on alpha < = 0.05.

^1^ ACR log-transformed; one percent change (in the independent variable) would yield a percentage change in ACR in models where the independent variable is log-transformed.

Total cholesterol and LDL cholesterol levels were significantly negatively associated with urinary ACR in the linear regression models in women.

### Longitudinal study

**Figure 2:**
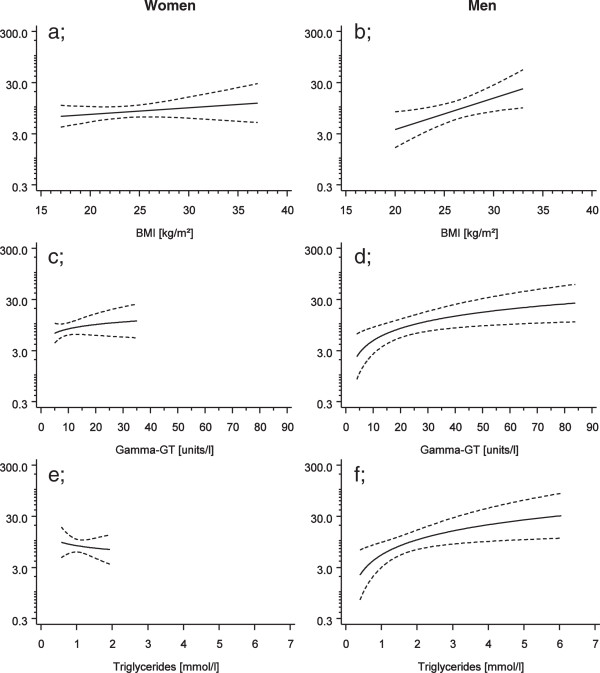
**Predicted mean ACR (mg/g) after 25 years of follow-up (baseline age 45 years) with 95% confidence bands by selected metabolic parameters.** Women presented on the left **(a, c, e)**, men on the right **(b, d, f)**.

The results of the longitudinal study are depicted in Table 3 (eGFR) and Table 4 (ACR). In men a consistent and significant negative association was found between BMI over all the time periods and current eGFR (Figure 2b). In addition, blood glucose levels measured >20 years before were negatively associated with eGFR.

**Table 3:** Longitudinal associations between anthropometric/ blood parameters and eGFR by sex

	**Previous parameters (over 20 years ago)**	**Previous parameters (10 to 20 years ago)**	**Previous parameters (5 to 10 years ago)**	**Current parameters (RENVOR)**
	**Beta (95% CI)**			
	**Women**			
N	35	35	35	35
Follow-up [years], median (Q1, Q3)	24.7 (23.5, 25.2)	15.0 (14.1, 15.4)	7.5 (7.1, 7.9)	-
BMI [kg/m^2^]	0.47 (−0.75, 1.69)	0.02 (−0.82, 0.87)	−0.03 (−0.78, 0.72)	0.35 (−0.42, 1.11)
Systolic blood pressure [mmHg]	0.21 (−0.04, 0.47)	* 0.22 (0.03, 0.42)	0.14 (−0.10, 0.37)	-
Log(Glucose) [mmol/l]	−2.99 (−19.89, 13.91)	−4.30 (−30.30, 21.69)	10.43 (−13.69, 34.56)	-
Total cholesterol [mmol/l]	1.04 (−5.91, 8.00)	1.07 (−4.83, 6.96)	−2.31 (−8.43, 3.80)	−2.71 (−8.03, 2.61)
Log(Triglycerides) [mmol/l]	2.90 (−16.61, 22.41)	−2.17 (−11.50, 7.17)	2.50 (−8.43, 13.43)	−0.91 (−11.82, 9.99)
Log(Gamma-GT) [units/l]	4.00 (−6.60, 14.59)	−4.37 (−12.79, 4.05)	−2.85 (−9.51, 3.81)	* -4.41 (−8.81, −0.00)
	**Men**			
N	42	42	42	42
Follow-up [years], median (Q1, Q3)	24.3 (21.9, 25.3)	14.9 (14.1, 15.9)	7.4 (6.9, 8.1)	-
BMI [kg/m^2^]	* -0.99 (−1.75, −0.24)	* -0.78 (−1.39, −0.17)	* -1.05 (−1.62, −0.48)	* -0.79 (−1.40, −0.19)
Systolic blood pressure [mmHg]	0.05 (−0.11, 0.21)	−0.14 (−0.30, 0.02)	0.02 (−0.13, 0.16)	-
Log(Glucose) [mmol/l]	* -9.70 (−17.41, −1.99)	−2.63 (−10.27, 5.01)	−5.33 (−16.18, 5.52)	-
Total cholesterol [mmol/l]	−2.04 (−4.20, 0.11)	−1.28 (−3.76, 1.20)	−1.81 (−4.45, 0.82)	−0.76 (−3.32, 1.81)
Log(Triglycerides) [mmol/l]	−4.29 (−9.51, 0.93)	−0.81 (−5.30, 3.68)	−4.02 (−8.83, 0.80)	−3.48 (−9.39, 2.43)
Log(Gamma-GT) [units/l]	−2.13 (−6.24, 1.97)	−0.78 (−5.62, 4.05)	−1.07 (−5.12, 2.98)	0.67 (−3.13, 4.47)

Models are adjusted for age.

* Significant on alpha < = 0.05.

**Table 4:** **Longitudinal associations between anthropometric/ blood parameters and ACR**^**1 **^**by sex**

	**Previous parameters (over 20 years ago)**	**Previous parameters (10 to 20 years ago)**	**Previous parameters (5 to 10 years ago)**	**Current parameters (RENVOR)**
	**Beta (95% CI)**			
	**Women**			
N	34	34	34	34
Follow-up [years], median (Q1, Q3)	24.7 (24.0, 25.2)	14.9 (14.1, 15.3)	7.5 (7.1, 7.9)	-
BMI [kg/m^2^]	0.03 (−0.03, 0.09)	0.02 (−0.02, 0.06)	0.00 (−0.03, 0.04)	0.00 (−0.04, 0.04)
Systolic blood pressure [mmHg]	−0.00 (−0.02, 0.01)	0.00 (−0.01, 0.01)	−0.00 (−0.01, 0.01)	-
Log(Glucose) [mmol/l]	* 0.91 (0.09, 1.73)	0.13 (−1.18, 1.44)	0.53 (−0.71, 1.77)	-
Total cholesterol [mmol/l]	−0.09 (−0.46, 0.27)	−0.05 (−0.35, 0.24)	* -0.39 (−0.67, −0.11)	* -0.28 (−0.54, −0.02)
Log(Triglicerides) [mmol/l]	−0.27 (−1.27, 0.74)	0.06 (−0.42, 0.54)	−0.17 (−0.72, 0.38)	0.20 (−0.35, 0.75)
Log(Gamma-GT) [units/l]	0.28 (−0.26, 0.82)	0.07 (−0.37, 0.51)	0.01 (−0.33, 0.35)	0.02 (−0.25, 0.29)
	**Men**			
N	42	42	42	42
Follow-up [years], median (Q1, Q3)	24.3 ( 22.8, 25.3)	14.9 (14.1, 15.9)	7.4 (6.9, 8.1)	-
BMI [kg/m^2^]	* 0.14 (0.03, 0.25)	0.08 (−0.02, 0.18)	0.09 (−0.00, 0.19)	0.06 (−0.04, 0.16)
Systolic blood pressure [mmHg]	−0.01 (−0.03, 0.01)	0.01 (−0.01, 0.04)	* 0.03 (0.01, 0.05)	-
Log(Glucose) [mmol/l]	0.23 (−0.83, 1.29)	* 2.27 (1.38, 3.16)	* 2.19 (0.71, 3.68)	-
Total cholesterol [mmol/l]	0.05 (−0.28, 0.38)	−0.01 (−0.38, 0.37)	−0.33 (−0.72, 0.06)	−0.03 (−0.43, 0.36)
Log(Triglicerides) [mmol/l]	* 0.98 (0.26, 1.70)	* 0.95 (0.34, 1.55)	* 0.91 (0.22, 1.59)	0.67 (−0.23, 1.57)
Log(Gamma-GT) [units/l]	* 0.79 (0.24, 1.34)	* 0.72 (0.03, 1.41)	* 0.65 (0.07, 1.22)	0.43 (−0.13, 1.00)

Models are adjusted for age.

* Significant on alpha <0.05.

^1^ ACR log-transformed; one percent change (in the independent variable) would yield a percentage change in ACR in models where the independent variable is log-transformed.

In women blood glucose measured at the time point over 20 years ago was positively associated with ACR. Blood glucose levels at later time points were also associated with ACR, however without statistical significance. In men, GGT and triglycerides were the most consistent predictor of increased ACR, with statistically significant associations in all previous observation periods (Figures 2c, 2e), whereas only the measurement made 20 years previously showed a significant association between BMI and current ACR. Serum glucose concentrations measured in the observation periods between 5 and 20 years were also strongly positively associated with current urinary ACR. In men systolic blood pressure measured 5–10 years before showed a positive association with current ACR. Sensitivity analyses including also individuals who had measurements in any but not in each of the time intervals revealed comparable patterns (data not shown).

An increasing number of abnormal metabolic factors was related to a decreasing eGFR and an increase of ACR (Table 5). Estimated GFR showed a stronger inverse association with the number of pathological metabolic factors in men than in women and was strongest for men in the most recent observation period (per one pathological factor: -2.81 95% CI −4.59;-1.03). The interaction with gender reached statistical significance (p-value 0.020). ACR also showed the strongest association in the most recent observation period in men (0.37 95% CI 0.08;0.65) and was statistically significantly different in women (p-value 0.041).

**Table 5:** **Association of sum score of pathologic values for BMI, systolic blood pressure, glucose, cholesterol, triglycerides and GGT with eGFR and ACR**^1^

	**>20 years ago**	**10 to 20 years ago**	**5 to 10 years ago**	**overall**
	**Sum score of pathologic values, median (Q1, Q3)**			
Women	1 (1, 2)	2 (1, 3)	2 (1, 3)	2.00 (1.33, 2.33)
Men	2.5 (1, 3)	2 (1, 3)	2 (1, 3)	2.33 (1.33, 3.33)
	**Change in eGFR (95%CI) for one additional pathologic value**			
Women	3.01 (−1.28, 7.28)	0.83 (−3.84, 5.49)	1.44 (−2.39, 5.26)	2.75 (−2.51, 8.00)
Men	−1.99 (−3.66, −0.31)	−1.27 (−3.17, 0.64)	−2.81 (−4.59, −1.03)	−2.62 (−4.62, −0.61)
Sex interaction, p-value	0.009	0.241	0.020	0.016
	**Change in log(ACR) (95%CI)**^**1**^**)for one additional pathologic value**			
Women	0.11 (−0.11, 0.33)	0.11 (−0.13, 0.34)	0.01 (−0.19, 0.20)	0.10 (−0.16, 0.37)
Men	0.29 (0.03, 0.55)	0.36 (0.09, 0.64)	0.37 (0.08, 0.65)	0.44 (0.14, 0.74)
Sex interaction, p-value	0.316	0.161	0.041	0.101

Models are adjusted for age.

^1^ ACR log-transformed; one additional pathological variable would yield a (100*β) percentage change in ACR.

## Discussion

Chronic kidney disease is increasingly recognized as a public health problem affecting more than 10% of individuals in population-based studies [9]. To bring the CKD epidemic under control it is highly desirable to early identify modifiable risk factors that can predict future CKD. However, the development of risk models for incident CKD is still in its infancy [10]. Therefore, long-term observational studies aimed at identifying anthropometric, metabolic or genetic risk factors that allow a more precise prediction of incident CKD are urgently awaited. Our study differs in some aspects from previous studies in this area [2,11-19]. We exclusively focused on patients at high risk for CKD. We performed a retrospective analysis by linking current data and data obtained at previous health examinations at regular time intervals dating back 25 years. According to the recent KDIGO definition of CKD, both eGFR and albuminuria should be reported to classify the CKD stage [20]. At an eGFR above 60 ml/min/1.73 m^2^ another renal abnormality, in most instances albuminuria, has to be present for the diagnosis of CKD. In our study the prevalence of kidney disease by means of combination of eGFR <60 ml/min/1.73 m^2^ and ACR >30 mg/g is low. Therefore we did not set these cut-offs for eGFR or ACR for our analysis, but instead used eGFR and ACR as continuous variables.

The most consistent finding in our long-term study was that higher BMI is associated with lower eGFR and greater ACR in men. Predicted mean eGFR after 25 years of follow-up in men decreased by 0.99 ml/min/1.73 m^2^ and ACR increased by 14% per 1 kg/m^2^ increase in BMI indicating a clinically relevant impairment of renal function. BMI was the only parameter associated with eGFR in the long-term as well as in the cross-sectional analyses. In the RENVOR study the median (Q1, Q3) BMI in men was 29.4 (26.1, 32.9) kg/m^2^ which is consistent with a high risk population. However, in the previous health examinations 20 years ago the median BMI in men was much lower (men 25.7 (23.3, 28.7) kg/m2). Over 25 years BMI increased on average 3.97 kg/m^2^ in men and 2.39 kg/m^2^ in women. The patient subset with previous examinations had slightly lower median current BMI than the RENVOR sample. However, the median WHR did not differ substantially.

An association between high BMI and obesity and CKD has been reported in several previous population-based studies with rather long follow-up [2,12,15,17,18]. In those studies CKD was defined as eGFR ≤60 ml/min/1.73 m^2^ or dipstick-positive proteinuria. Our study suggests that the associations between BMI and eGFR or albuminuria are present even in near normal ranges of these markers of renal function. A very recent study found a doubling of the risk for CKD defined as eGFR <60 ml/min/1.73 m^2^ and a 70% increase in the risk for albuminuria in individuals over 60 years of age who were overweight in adolescence or early adulthood [21]. A recent development of overweight was not associated with renal disease. This study, however, did not report on possible differences between men and women.

Whether obesity is linked to CKD by its consequences such as diabetes or hypertension, or whether other pathways directly lead to CKD in obese individuals is controversial [2,18]. Other authors have suggested that increased WHR may be a better marker than BMI for subsequent kidney disease, because WHR reflects central obesity [19]. The association between WHR and BMI and their link to cardiovascular disease are modified by sex. Men with a high BMI usually have central obesity and a high WHR, and therefore WHR is not a better marker for cardiovascular risk in men. In women, however, WHR seems to be a better marker for CVD risk as compared to BMI [22]. Whether the same phenomenon also holds true for renal risk prediction is unknown to date. If true, this could explain our finding of an association between BMI and low eGFR and increased albuminuria in men, but not in women. The statistically non-significant protective association between BMI and eGFR in our study may also be related to sex-specific body fat distribution. Unfortunately, WHR was not determined at previous VHM&PP investigations, and therefore we are unable to determine whether WHR would have predicted CKD in women or not.

Central obesity is included in the definition of the metabolic syndrome, which in addition is defined by the presence of hypertension, impaired glucose tolerance, high triglycerides and low HDL cholesterol. There is no single accepted definition of MetS and also some controversy regarding its existence [23]. Nevertheless all existing definitions include indicators of insulin resistance, lipid abnormalities, blood pressure and obesity [24,25]. An association between the metabolic syndrome and CKD has been described in a number of studies [5,13,16,26,27]. This association increases with the number of given components of the metabolic syndrome [5]. Consistently, we found in our study a decrease in eGFR of 1.99 ml/min/1.73 m^2^ for each additional pathological metabolic factor present 20 years before (BMI, blood pressure, glucose, triglycerides, total cholesterol). Of note, we found that high fasting glucose and triglycerides were predictive of higher albumin excretion in men and women in our long-term study. A relationship between the metabolic syndrome and albuminuria in men but not women has also been described by others, albeit without a significance for fasting glucose, lipids or BMI [16]. The follow-up of that study, however, was only six years and therefore considerably shorter than our investigation.

One finding of special interest in our study is the consistent association between GGT levels and albuminuria in men. Elevated serum GGT has been shown to be predictive of CVD events, diabetes, hypertension, the metabolic syndrome, cancer and mortality [6,28,29]. Whether high GGT levels are also a marker of incident kidney disease is less clear. Two cross-sectional studies using National Health and Nutrition Examination Survey data came to different conclusions. Whereas one study found a strong association between GGT and CKD [30], the other study found no such relation [31]. An investigation with a 3-year follow-up described an association between GGT and the development of CKD (defined as eGFR < 60 ml/min/1.73 m^2^) in men without hypertension or diabetes [32]. Another study over a 15-year period found that GGT was predictive of microalbuminuria in individuals with diabetes or hypertension [33]. These results together with our study would suggest that high serum GGT levels are a good predictor of albuminuria in high-risk patients, particularly in men. The mechanisms that link GGT to CVD, hypertension, diabetes and albuminuria are unclear at present. A recent study found an association between GGT levels in the upper normal range and components of the metabolic syndrome (obesity, high blood pressure, and low LDL cholesterol), even in children and adolescents [34]. High GGT may be a marker of non-alcoholic fatty liver disease, which is associated with the metabolic syndrome. In particular, big GGT (b-GGT), a large aggregate of proteins most likely representing exosomes released from cells, is found to be elevated in non-alcoholic fatty liver disease [35]. Whether this specific b-GGT fraction is also associated with other components of the metabolic syndrome is unclear at present. GGT metabolizes reduced glutathione and may be a marker of oxidative stress [36]. In addition, high GGT has been linked to inflammation [37].

Limitations of our study include the lack of data on covariates, which may have influenced risk estimates. However, additional adjustment for smoking status did not substantially change the associations. The number of subjects included in the 25-year observation is rather small and limits the statistical power of this explorative study. However, sensitivity analyses including individuals who had examinations in any of the time periods revealed virtually similar results and the associations across time are consistent. Unfortunately, eGFR and albuminuria were not determined at the prior investigations. We do not have information on medication or comorbidities, but as hypertension, diabetes or CVD were diagnosed at a median time of six to eight years before the cross-sectional investigation, almost all individuals were most likely healthy, at least when examined over 20 years ago. We did not have information on lipid sub-fractions, instead we used total cholesterol. However, we found that an increasing number of metabolic aberrations are associated inversely with eGFR and positively with ACR in men. There is also evidence that other metabolic factors such as GGT could be involved in the pathogenesis of CKD [7]. This marker of oxidative stress could be investigated in our study.

The major strength of this study is the very long follow-up of more than 20 years and the complete datasets for all patients at the specified time periods.

## Conclusion

In conclusion, our results provide further evidence that anthropometric and metabolic factors influence eGFR and urinary albumin excretion during more than 20 years of follow-up. The predictive value of these factors is modified by sex. BMI is the most consistent long-term risk factor in men. In addition, glucose, triglycerides and GGT seem to be associated with renal function especially albuminuria.

## Abbreviations

(CKD): Chronic kidney disease; (RENVOR): Renal disease in vorarlberg; (VHM&PP): Vorarlberg health monitoring & prevention program; (eGFR): Estimated glomerular filtration rate; (ACR): Urinary albumin creatinine ratio; (GGT): Gamma-glutamyltransferase; (BMI): Body mass index; (MetS): Metabolic syndrome; (LDL): Low density lipoprotein; (HDL): High density lipoprotein; (CRP): C-reactive protein; (WHR): Waist-to-hip ratio; (CVD): Cardiovascular disease.

## Competing interests

The authors declare that they have no competing interests.

## Authors’ contribution

KL, HC conception of the study and data collection; GN, RP, EZ data analyses; GN wrote the first draft, which was refined by contributions of LK, EZ, AP. All authors were involved in the interpretation of the data, read and approved the final manuscript.

## Pre-publication history

The pre-publication history for this paper can be accessed here:

http://www.biomedcentral.com/1471-2369/14/177/prepub
